# Genetics of resistance to *Zymoseptoria tritici* and applications to wheat breeding

**DOI:** 10.1016/j.fgb.2015.04.017

**Published:** 2015-06

**Authors:** James K.M. Brown, Laëtitia Chartrain, Pauline Lasserre-Zuber, Cyrille Saintenac

**Affiliations:** a John Innes Centre, Norwich Research Park, Colney, Norwich NR4 7UH, England, United Kingdom; b INRA, UMR 1095, Genetics, Diversity and Ecophysiology of Cereals, F-63100 Clermont-Ferrand, France; c UBP, UMR 1095, Genetics, Diversity and Ecophysiology of Cereals, F-63100 Clermont-Ferrand, France

**Keywords:** Durable resistance, Gene-for-gene relationship, Genetic mapping, Plant breeding, Quantitative trait locus (QTL), *Septoria tritici* blotch

## Abstract

This paper reviews current knowledge about genes for resistance to *Septoria tritici* blotch (STB) of wheat, caused by *Zymoseptoria tritici* (formerly *Mycosphaerella graminicola*). These genes can be placed into two classes, although a few may have characteristics of both classes. Qualitative resistance is controlled by genes which control large fractions of genetic variation, 21 of which have been discovered and mapped so far. Most of them have been shown to be genotype-specific, being effective against the minority of *Z*. *tritici* isolates which are avirulent, and *Stb6* has been shown to control a gene-for-gene relationship. Most qualitative resistances are unlikely to be durable and some formerly effective genes have been overcome by the evolution of pathogen virulence. Quantitative resistance is generally controlled by genes with small-to-moderate effects on STB. They have generally weaker specificity than qualitative genes and have provided more durable resistance. 89 genome regions carrying quantitative trait loci (QTL) or meta-QTL have been identified to date. Some QTL have been mapped at or near loci of qualitative genes, especially *Stb6*, which is present in several sources of resistance. Another gene of particular interest is *Stb16q*, which has been effective against all *Z*. *tritici* isolates tested so far. In addition to resistance, the susceptibility of wheat cultivars to STB can also be reduced by disease escape traits, some of which may be undesirable in breeding. The fundamental requirements for breeding for STB-resistance are genetic diversity for resistance in wheat germplasm and a field trial site at which STB epidemics occur regularly and effective selection can be conducted for resistance combined with other desirable traits. If these are in place, knowledge of resistance genes can be applied to improving control of STB.

## Highlights

• We review the genetics of wheat resistance to *Septoria tritici* blotch, with a map of known genes.
• Qualitative resistance is usually monogenic, genotype-specific and non-durable.
• Quantitative resistance is generally polygenic with low specificity and greater durability.
• Major requirements for resistance breeding are diverse germplasm and field sites with severe Septoria.

## 1 Types of resistance to *Septoria tritici* blotch

Resistance to *S. tritici* blotch (STB; caused by *Zymoseptoria tritici*, formerly *Mycosphaerella graminicola*) became a significant target trait in wheat breeding much more recently than diseases such as the rusts and powdery mildew. The potential threat posed by STB was drawn to international attention by a very damaging epidemic in North Africa in 1968–1969, which followed the introduction of semi-dwarf wheat cultivars and increased use of artificial fertiliser (Saari and Wilcoxson, 1974). Subsequently, STB increased in importance, particularly in semi-dwarf cultivars given high rates of nitrogen fertiliser (Wiese, 1987) and is now a potentially damaging disease throughout the temperate regions (HGCA, 2012; O’Driscoll et al., 2014; Fones and Gurr, 2015). Early work on genetics (reviewed by Goodwin, 2007) focussed on the discovery of sources of resistance for breeding and on cultivar-by-isolate interaction but substantial progress has been made in the last 20 years in the genetics of resistance. This is giving breeders a deeper understanding of effective approaches to improving resistance (Torriani et al., 2015) and will allow resistance genes to be isolated and their functions revealed.

As in many other plant diseases, wheat has essentially two types of resistance to STB, as shown in a large study of 236 wheat cultivars grown in the UK in the 1990s and their progenitors (Arraiano and Brown, 2006; Arraiano et al., 2009). *Qualitative resistance* is strong and is usually controlled by major genes with a large effect. These genes are generally effective against avirulent pathogens but not against other, virulent isolates. Their pattern of interaction with *Z*. *tritici* accords with the gene-for-gene relationship, as has been demonstrated for *Stb6* (Brading et al., 2002). *Quantitative resistance*, by contrast, has a partial phenotype and is controlled by several-to-many genes with moderate-to-small effects. In many instances but not always, it is effective against all *Z*. *tritici* genotypes. Even though STB is almost always scored as a quantitative trait or at least on an ordinal scale, segregation of a qualitative gene can give rise to a large difference between two groups of progeny, resistant and susceptible (e.g. *Stb6*; Brading et al., 2002). This can be obscured, however, by segregation of minor genes which alter the level of STB symptoms and lead to intermediate phenotypes.

In certain cultivar-by-isolate interactions, adult-plant responses to *Z*. *tritici* do not necessarily reflect responses of seedlings to the pathogen (Kema and van Silfhout, 1997; Chartrain et al., 2004a). Many genotype-specific, qualitative resistances are independent of growth stage (Kema and van Silfhout, 1997; Arraiano et al., 2001a; Brown et al., 2001; Grieger et al., 2005) whereas the expression of partial resistance may depend on the plant’s growth stage (Chartrain et al., 2004a). *Stb17* is an example of a gene with a quantitative effect on disease which is expressed in adult plants but not seedlings (Tabib Ghaffary et al., 2012), while genes on chromosome arm 5BS of Hobbit sib increased susceptibility only in adult plants (Arraiano et al., 2007a).

**Fig. 1:**
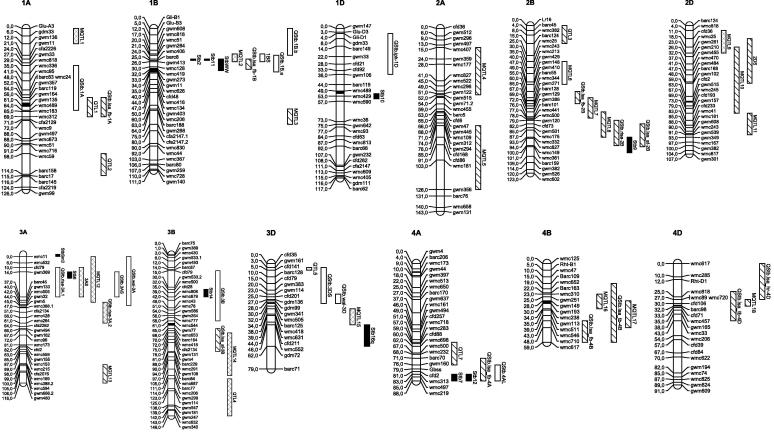

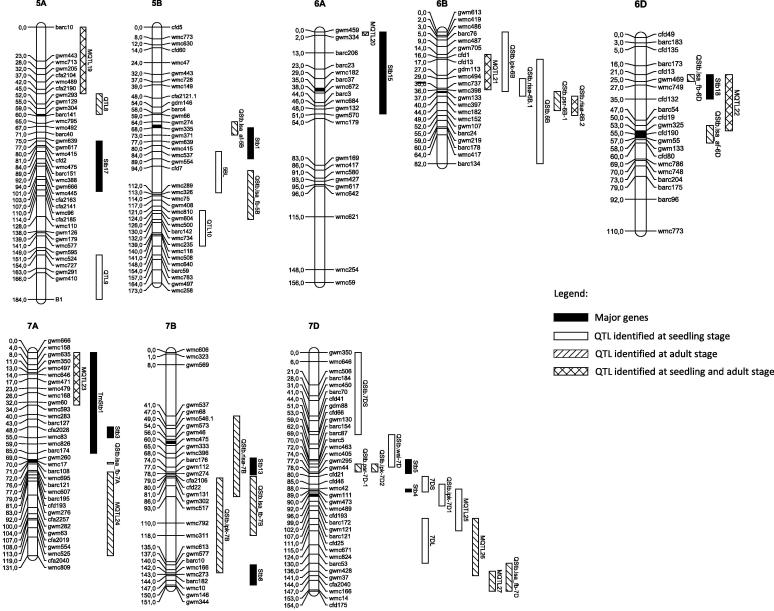
Location in the wheat genome of major genes, QTL and meta-QTL involved in resistance to *Septoria tritici* blotch. Loci have been projected on the simplified SSR consensus map of Somers et al. (2004). Five QTL from Table S1 were not included in the map due to a lack of shared markers between the original paper and the consensus map. Solid bars represent major genes (see Section 1 and Table 1) and other bars patterns indicate QTL identified at different plant growth stages.

In this review, we survey the genes for STB-resistance reported to date (Fig. 1), including both qualitative (Table 1) and quantitative resistance (Table S1), beginning with the first genes to be named, *Stb1*, *Stb2* and *Stb3* (Wilson, 1985). Earlier reports of sources and genetics of resistance were summarised by Kema et al. (1996a, 1996b), Goodwin (2007) and Raman and Milgate (2012). We also describe how knowledge of the genetics of resistance can be applied to wheat breeding.

## 2 Methods of studying resistance

As with genetic analysis of any trait, study of STB-resistance requires a method of scoring the phenotype which can be applied to large populations. Tests can be conducted at the seeding stage, with inoculation typically when seedlings are two weeks old. Both whole-seedling (Brading et al., 2002) and detached leaf assays (Arraiano et al., 2001a) are used, and require conditions with high relative humidity. They generally take around four weeks following inoculation. The advantage of using whole seedlings is that many plants can be tested but a disadvantage, especially in greenhouse trials, is that environmental conditions may not be strictly controlled, which can affect the development of disease (Arraiano et al., 2001a). Detached-leaf tests are particularly suitable when many isolates are to be tested, although this can also be done in a greenhouse (Kema et al., 1996a). Alternatively, plants can be tested at the adult stage in the glasshouse (Adhikari et al., 2003) or field (Kema and van Silfhout, 1997). Given the genotype-specificity of most qualitative resistances (see below), identification of single genes in field conditions requires plants to be inoculated with a *Z*. *tritici* isolate at a dose sufficient to make contamination by natural inoculum comparatively negligible (Kema and van Silfhout, 1997; Brown et al., 2001). The expression of symptoms following inoculation in adult plants proceeds at a broadly similar rate as in seedlings. When genetic analysis is conducted on naturally-infected trials, the genes identified are effective against the current local *Z*. *tritici* population. This is generally relevant to the practice of plant breeding but may have implications for repeatability if there is polymorphism at avirulence loci corresponding to segregating resistance genes.

In most genetic analyses of STB-resistance, the phenotype studied is formation of pycnidia, the asexual fruiting bodies of *Z*. *tritici* which form within necrotic tissue on the leaf. In this case, the data are fractions of leaf area covered by necrotic lesions bearing pycnidia. Some studies have reported other phenotypes in addition to pycnidium formation, including total necrotic leaf area with or without pycnidia, latent period and disease progress. These traits are scored visually, either by eye or, more recently, by computer-aided image analysis (Stewart and McDonald, 2014).

## 3 Qualitative resistance

Please refer to Table 1 for details of genes for STB-resistance in bread wheat (*Triticum aestivum*) and to Fig. 1 for their locations.

### 3.1 Stb1, Stb2 and Stb3

*Stb1*, *Stb2* and *Stb3* were the first genes for STB-resistance to be named (Wilson, 1985). Before then, it was generally thought that resistance to *Z*. *tritici* was a quantitative, polygenic trait. Although quantitative resistance is indeed considerably more important than qualitative resistance in wheat breeding, the discovery that significant amounts of resistance can be controlled by major genes opened the way to genetic analysis of STB and may offer an opportunity to improve resistance by ‘stacking’ or ‘pyramiding’ several *Stb* genes (Chartrain et al., 2004b; see Section 6.1).

*Stb1*, *Stb2* and *Stb3* have been mapped to chromosome arms 5BL (i.e. the long arm of chromosome 5B; Adhikari et al., 2004a), 1BS (the short arm of 1B; Liu et al., 2013) and 7AS (Goodwin and Thompson, 2011) respectively. *Stb2* and *Stb3* were originally mapped to chromosomes 3BS and 6DS respectively (Adhikari et al., 2004b) but those locations were corrected for the reasons given in the subsequent papers. *Stb2* was found to map to the same region of 1BS as *Stb11* (Chartrain et al., 2005a) but no test of allelism of the two genes has yet been done.

The sources of *Stb1*, *Stb2* and *Stb3* – cvv. Bulgaria 88, Veranopolis from Brazil, and Israel 493 respectively – all have *Stb6* in addition (Chartrain et al., 2005b). The resistance of Bulgaria 88 was described as durable by Adhikari et al. (2004a) but it is not known if this refers to *Stb1*, *Stb6* or both.

### 3.2 Stb4

*Stb4* in cv. Tadinia was the first gene to be identified by controlled inoculation with a single isolate of *Z*. *tritici*, CA30 from California (Somasco et al., 1996). It was subsequently mapped to chromosome arm 7DS (7DS; Adhikari et al., 2004c), close to the locus of *Stb5* (Arraiano et al., 2001b). Again, the allelism of *Stb4* and *Stb5* has not been tested.

### 3.3 Genes from synthetic hexaploid wheat: Stb5, Stb8, Stb16q and Stb17

The first gene for STB-resistance to be mapped was *Stb5* (Arraiano et al., 2001b), which originated from a highly resistant synthetic hexaploid line, Synthetic 6x, derived from *Triticum dicoccoides* (AABB genomes) and *Triticum tauschii* (also known as *Aegilops squarrosa*; DD). *Stb5* mapped to the pericentromeric region of chromosome arm 7DS, close to where *Stb4* was mapped subsequently (Adhikari et al., 2004c). The mapping work used *Z*. *tritici* isolate IPO94269 from The Netherlands but *Stb5* conferred resistance to all but one of the isolates tested. The location of *Stb5* was greatly facilitated by the use of precise cytogenetic stocks (Arraiano et al., 2001b; Simon et al., 2001), which form a unique resource for wheat genetics to identify chromosomes carrying genes for traits of interest.

Synthetic hexaploids are a rich source of qualitative genes for resistance to STB and other diseases. *Stb8* was identified in another synthetic line, W7984, bred by CIMMYT (the International Maize and Wheat Improvement Centre). It conferred resistance to an isolate from the USA and mapped to the long arm of chromosome 7B (Adhikari et al., 2003).

Two further genes, *Stb16q* and *Stb17*, were discovered in the synthetic hexaploid line M3 (Tabib Ghaffary et al., 2012). *Stb16q* on chromosome 3DL was designated as a quantitative (*q*) locus because it was not possible to determine if there was indeed a single gene at the locus. However, it controlled a high proportion of variation in necrotic leaf area, leaf area bearing pycnidia and latent period, and, alone among major *Stb* genes reported so far, conferred resistance at the seedling stage to all *Z*. *tritici* isolates tested, of which there were 20. It may be better regarded as a type of qualitative resistance. *Stb17* on 5AL was detected only at the adult-plant stage and was less potent than *Stb16q*. The phenotype of *Stb17* may fall between the qualitative and quantitative classes.

### 3.4 Stb6

The only qualitative gene for STB-resistance which has been shown to control a gene-for-gene relationship is *Stb6*, at the distal end of the short arm of chromosome 3A (Brading et al., 2002). This gene, which confers resistance to a Dutch *Z*. *tritici* isolate, IPO323, was first identified in the UK cvv. Flame and Hereward.

*Stb6* is an especially notable gene as it was found to be present in most of the well-known sources of STB-resistance studied previously. Analysis of alleles of a simple-sequence repeat (SSR or microsatellite), *Xgwm369*, closely linked to *Stb6*, allied to analysis of wheat breeding pedigrees indicated that *Stb6* had been introduced on at least six separate occasions into modern European germplasm, and was also present in Chinese Spring, a selection from a landrace which has been widely used in genetic studies of wheat (Chartrain et al., 2005b). It is the second most frequent *Stb* gene in European wheat, present in about 15% of cultivars tested (Arraiano and Brown, 2006).

At least five analyses of quantitative trait loci (QTL; Eriksen et al., 2003; Zwart et al., 2010; Tabib Ghaffary et al., 2011; Kelm et al., 2012; Goudemand et al., 2013) as well as an association genetic analysis (Kollers et al., 2013) have mapped field resistance to STB close to the *Stb6* locus. A study of a large panel of UK and continental European cultivars found *Stb6* to be associated with a reduction in STB symptoms in field conditions (Arraiano et al., 2009). This is consistent either with a minor gene for partial resistance to STB being closely linked to *Stb6* or with *Stb6* itself having a residual effect on field resistance even though virulence to *Stb6* is almost fixed in the European population of *Z*. *tritici* (J.K.M.B., unpublished data).

Avirulence (AVR) to *Stb6* was shown to be controlled by a single gene in *Z*. *tritici* IPO323 in a cross with IPO94269 in which virulence to several cultivars and breeding lines co-segregated (Kema et al., 2000; Brading et al., 2002). As resistance to IPO323 maps to the *Stb6* locus in all these cultivars (Brading et al., 2002; Chartrain et al., 2005b), it is concluded that *Stb6* is present in all of them and that it corresponds to a single *AVR* gene in IPO323.

### 3.5 Stb7 and Stb12

*Stb7* on chromosome 4AL was first identified in cultivar ST6 as conferring resistance to the Canadian isolate MG2. It was mapped close to SSR locus *Xwmc313* (McCartney et al., 2002, 2003). A gene for resistance to the Uruguayan isolate IPO87019 which mapped in the same location in the Portuguese line TE9111 (later released as cv. Nabão) was thought to be *Stb7*, an allele of *Stb7* or a closely linked gene (Chartrain et al., 2005a). As the resistance gene in TE9111 was mapped by QTL analysis a precise location for the gene could not be achieved.

The CIMMYT breeding Kavkaz-K4500 L.6.A.4 (KK) carries *Stb12*, which is closely linked to *Stb7* on chromosome 4AL. *Stb12* provides resistance to isolate Isr398 from Israel but not to IPO87019 (Chartrain et al., 2005c). It was mapped by QTL analysis and was closer to *Xgwm219* than to *Xwmc313*, which are ∼3cM apart. Of the 94 single-seed descent progeny of the cross of KK with the Isr398-susceptible cv. Shafir, four were resistant to ISR398 but susceptible to IPO97019, demonstrating the existence of two genes in that region. This is an example of *Stb* genes being clustered, a common feature of genes involved in plant defence.

### 3.6 Other qualitative genes in bread wheat

*Stb9* on chromosome 2BL was mapped in the spring wheat cvv. Courtot and Tonic (Chartrain et al., 2009). It confers resistance to the Dutch isolate IPO89011.

*Stb10* was also discovered in KK. Like *Stb5*, it conferred resistance to IPO94269 but it was clearly a different gene, located near the centromere of chromosome 1D (Chartrain et al., 2005c).

*Stb11* on chromosome 1BS was identified and mapped in TE9111 and reported to confer resistance to isolate IPO90012 from Mexico (Chartrain et al., 2005a) but it may be widespread in global spring wheat breeding. When remapped, *Stb2* was located close to or at the *Stb11* locus (Liu et al., 2013). *StbWW*, identified in three populations in Australia, was also mapped on chromosome arm 1BS at or near *Stb11* (Raman et al., 2009). These genes may all be *Stb11*, which may have spread in global wheat breeding by the movement of elite breeding lines from CIMMYT.

*Stb13* on chromosome 7BL and *Stb14* on 3BS were discovered in the Canadian cv. Salamouni (McCartney et al., 2002; Cowling, 2006). Both genes conferred resistance to MG2, like *Stb7*, while *Stb13* also provided resistance to MG96-36 (Cowling et al., 2004; Cowling, 2006). Salamouni also has a third gene, designated *StbSm3*, which maps close to the *Stb6* locus on chromosome 3AS but apparently distal to it (Cuthbert, 2011). No test of the allelism of *StbSm3* and *Stb6* has yet been conducted.

*Stb15* on chromosome 6AS was identified as providing resistance to the Ethiopian isolate IPO88004 (Arraiano et al., 2007b). It is very common in European winter wheat, present in about 60% of cultivars tested (Arraiano and Brown, 2006) but, unlike the other widespread gene, *Stb6*, it is not associated with resistance in field conditions (Arraiano et al., 2009).

*Stb18* on 6DS confers genotype-specific resistance in the French winter wheat cv. Balance (Tabib Ghaffary et al., 2011). It was expressed at the seedling stage but inconsistently in adult plants, being detected in one of two years of field trials of a population produced from Apache x Balance.

### 3.7 Qualitative resistance in durum wheat

Although STB is a severe disease of modern cultivars of durum wheat (*Triticum durum*), especially in North Africa, the genetics of STB-resistance in *T*. *durum* are poorly understood. In a search for sources of resistance in older, landrace cultivars, resistance to *Z*. *tritici* isolate Tun06 in a selection from the Agili landrace segregated as a single major gene (Ferjaoui et al., 2011). This gene was associated with AFLP markers but has not yet been assigned to a chromosome (Medini et al., 2014).

### 3.8 Resistance in Triticum monococcum

The diploid emmer wheat, *Triticum monococcum*, is highly resistant to *Z*. *tritici*. All accessions tested varied from very resistant to immune both to artificial resistance as seedlings and in five years of field trials. The genetics of resistance were studied in one accession, MDR043, and the gene *TmStb1* was mapped to chromosome 7A^m^S (Table 1; Jing et al., 2008).

## 4 Quantitative resistance

Please refer to Table S1 for details of these genes and to Fig. 1 for their locations.

### 4.1 QTL in bi-parental crosses

In field trials, resistance to STB generally appears as a quantitative trait, largely additive in nature with some dominance, controlled by an oligogenic or polygenic system with moderate to high heritability in both durum wheat (van Ginkel and Scharen, 1987, 1988; Berraies et al., 2014) and bread wheat (Danon and Eyal, 1990; Jlibene et al., 1994; Simon et al., 1998; and papers cited in Section 4 and Table S1). QTL for resistance to STB at both seedling and adult stages are distributed throughout the genome of wheat (Table S1a). To date, 167 QTL of resistance against STB have been detected in a total of nineteen bi-parental mapping populations. From seven of these populations, 27 meta-QTL, i.e. refined QTL from multiple individual QTL, have been identified, integrating 105 individual QTL (Goudemand et al., 2013; Table S1b). Of 89 regions identified, 62 QTL and 27 meta-QTL, 27 were detected at the seedling stage, 48 at the adult stage and 14 at both stages. They included genome regions involved in the control of necrosis, pycnidium development and disease progress estimated as area under the disease progress curve (AUDPC). Two minor QTL controlling latent period have also been identified (Tabib Ghaffary et al., 2011).

All chromosomes except 5D carry at least one QTL or meta-QTL for STB-resistance. Nineteen QTL or meta-QTLs co-localised with genes involved in plant height (*Rht*: reduced height), heading date (*Ppd*: photoperiod-insensitivity) or both, among which six mapped closely to the *Rht8* and *Ppd-D1* (2DS), *Rht-D1* (4DS), *Ppd-A1* (2AS) and *Rht-B1* (4BS). Three chromosome arms, 3BL, 6BS and 7DL, were especially involved in quantitative resistance to STB according to the number of QTLs identified. There are probably co-localisations with qualitative *Stb* genes for 22 QTL and 6 meta-QTL. QTL have frequently been mapped to the regions where *Stb6* (3AS), *Stb5*/*Stb4* (7DS) and *Stb11*/*Stb2*/*StbWW* (1BS) are located and less frequently to the regions of *Stb1* (5BL), *Stb9* (2BL), *Stb7* and *Stb12* (4AL), *Stb13* (7BL), *Stb14* (3BS) and *Stb18* (6DS). Except for eight QTL identified in synthetic hexaploid wheat (Simon et al., 2004a; Zwart et al., 2010) the chromosome substitution line ‘Chinese-Spring’ (*T*. *aestivum* subsp. *spelta* 7D) (Simon et al., 2010) and a line from the USA (Mergoum et al., 2013), all QTL listed in Table S1 originated from European germplasm.

### 4.2 Association genetics

Association mapping studies have highlighted the presence of many regions of the genome in cultivated wheat and landraces associated with resistance to STB, including both some previously associated with STB-resistance and some not. From spray-inoculated field trials conducted over two years, Kollers et al. (2013) detected 68 SSR significantly associated with adult resistance in a panel of 372 European lines. Nine loci were significantly associated with all phenotyping parameters. Association QTL mapped to the loci of *Stb1*, *4*, *6* and *8*, implying that these genes or alleles of them may be present in European cultivars. In addition, several traits related to STB-resistance mapped at or near QTL identified previously.

In a study of 1055 elite hybrids and their corresponding 87 parental lines trialled in two locations which either had natural infection or were inoculated by spraying with a mixture of isolates, Miedaner et al. (2013) identified eight single-nucleotide polymorphisms (SNP) associated with STB resistance. Although half the SNP were not genetically mapped, the others were located on chromosomes 1B, 2B, 5B and 6A. The 5B locus may represent *Stb1* or a QTL identified in the population Arina x Forno (Miedaner et al., 2012) and Steele-ND x ND 735 (Mergoum et al., 2013).

Finally, seven SNP at four loci were significantly associated with resistance in an association mapping study of a panel of 528 spring wheat landraces of worldwide origin phenotyped at the adult stage in growth chambers. These SNP mapped to chromosomes 3B, 6B and 7B and most likely relate to new resistance genes (Gurung et al., 2014).

### 4.3 Cytogenetics

In a cytogenetic analysis, the 5BS arm of Hobbit sib (Dwarf A) was found to promote susceptibility to STB in adult plants but not in seedlings. Formally, the data were also consistent with this chromosome arm carrying genes which suppress resistance but Hobbit sib has no known STB-resistance genes. The same chromosome arm has genes which increase resistance to yellow (stripe) rust and powdery mildew, which implies that there may be a trade-off between breeding for resistance to STB and to biotrophic fungi (Arraiano et al., 2007a).

## 5 Specificity of resistance

### 5.1 Specialisation of Z. tritici to host cultivars and species

Strong specificity in the interaction between cultivars of bread wheat (*T. aestivum*) and *Z*. *tritici* isolates was reported by Eyal et al. (1973) and confirmed in subsequent studies on seedlings (Ahmed et al., 1995; Ballantyne and Thomson, 1995; Kema et al., 1996a, 1996b; Arraiano et al., 2001a; Chartrain et al., 2004b; Grieger et al., 2005; Arraiano and Brown, 2006; Medini and Hamza, 2008; Czembor et al., 2011; Abrinbana et al., 2012) and adult plants (Kema and van Silfhout, 1997; Brown et al., 2001; Grieger et al., 2005). These interactions are akin to gene-for-gene relationships but this has only been demonstrated in *Stb6* resistance to IPO323 (Kema et al., 2000; Brading et al., 2002).

Specialisation of *Z*. *tritici* isolates to either *T*. *aestivum* or *T*. *durum* has been reported by some workers (Eyal et al., 1973; Kema et al., 1996a, 1996b and papers cited therein; Zhan et al., 2004) but not others (Eyal, 1999; Medini and Hamza, 2008). Cultivar-by-isolate specificity within *T*. *durum* has also been reported (Kema et al., 1996a, 1996b; Medini and Hamza, 2008; Ghaneie et al., 2012). In a cross of *aestivum*-adapted and *durum*-adapted isolates of *Z*. *tritici*, the *AvrStb6* locus for avirulence to *Stb6* (Brading et al., 2002) was associated with part of the variation in ability of progeny isolates to infect *T*. *durum*. This suggests that resistance of these two wheat species to inappropriate specialised forms of *Z*. *tritici* may be controlled in part by qualitative resistance genes (Ware, 2006).

Most *Z*. *tritici* isolates used in research on STB are virulent to almost all *Stb* genes although some isolates with more than one functional avirulence phenotype are known. IN95-Lafayette-1196-WW-1-4 was avirulent to both *Stb1* (Adhikari et al., 2004a) and *Stb4* (Adhikari et al., 2004c) while the Paskeville isolate was avirulent to *Stb2* and *Stb3* (Adhikari et al., 2004b). Among the isolates avirulent to *Stb5* in Synthetic 6x (Arraiano et al., 2001b) were IPO323, which is also avirulent to *Stb6* (Brading et al., 2002), IPO89011 (avirulent to *Stb9*: Chartrain et al., 2009), IPO94269 (avirulent to *Stb10*: Chartrain et al., 2005c) and IPO001, which is avirulent to some UK cultivars (Arraiano and Brown, 2006). MG2 was avirulent to *Stb7* (McCartney et al., 2002), *Stb13* and *Stb14* (Cowling, 2006). IPO323 may detect a second resistance gene in KK in addition to *Stb6* (Chartrain et al., 2005b). Otherwise, the high frequency of virulence implies that there is little obstacle to *Z*. *tritici* mutating to virulence on *Stb* genes which control gene-for-gene interactions.

### 5.2 Evolution of virulence

As in other diseases with a gene-for-gene system, the specificity of qualitative resistance to avirulent *Z*. *tritici* genotypes leads to selection for virulence (loss of avirulence). In Oregon, USA, such a ‘breakdown’ of resistance due to pathogen adaptation happened rapidly, with a catastrophic effect on disease control, in cv. Gene in the 1990s (Cowger et al., 2000) and more gradually in cv. Foote in the 2000s (Krenz et al., 2008). Cv. Gene is resistant to IPO323 and IPO94269, which are avirulent to *Stb6* and *Stb10* respectively (Chartrain et al., 2004b) but it is not known if either of these genes was the one which was overcome by the fungus. Kema and van Silfhout (1997) reported that the resistance of cv. Obelisk became less effective in The Netherlands during the 1980s. Virulence to *Stb4* evolved in *Z*. *tritici* in California at some time before 2000 (Jackson et al., 2000).

## 6 Breeding for resistance to *S. tritici* blotch

### 6.1 The use of qualitative STB-resistance in breeding

A superficially attractive option for breeding for resistance to STB, as in many other plant diseases, is to use qualitative genes with large effects on the pathogen. Synthetic hexaploid wheat may be a rich source of such genes (Arraiano et al., 2001b Adhikari et al., 2003; Dreisigacker et al., 2008; Tabib Ghaffary et al., 2012). A persistent difficulty in applying this strategy in plant breeding is that many (but not all) single genes which confer strong resistance conform to the gene-for-gene relationship, while most gene-for-gene resistances (but not all) are readily overcome by the target pathogens (Poland et al., 2008; Mundt, 2014).

Breeding for resistance to STB can benefit greatly from the long history of breeding crops to control other diseases. While reliance on qualitative genes may reduce STB in the short-term, this approach is unlikely to provide durable resistance. A relevant comparison is with the successive use of gene-for-gene resistances against powdery mildew of barley (Brown, 1994) or yellow (stripe) rust of wheat (Hovmøller and Justesen, 2007), a strategy which has been far from durable. The useful lifetime of qualitative genes can be extended by supporting them with high levels of ‘background’, usually durable, quantitative resistance (Palloix et al., 2009).

It is striking that some notable sources of STB-resistance, such as KK (Chartrain et al., 2004b, 2005c), Salamouni (Cowling, 2006; Cuthbert, 2011) and TE9111 (Chartrain et al., 2005a) have several qualitative resistances (Chartrain et al., 2004b). This suggests that ‘stacking’ or ‘pyramiding’ several *Stb* genes might improve the effectiveness of resistance, a strategy which has sometimes been effective in controlling crop diseases (Mundt, 2014). Against this, the fact that most known *Z*. *tritici* isolates are virulent to most *Stb* genes (Section 5.1) suggests that the resistance achieved by gene-stacking may not be durable.

### 6.2 Selection for durable resistance

Known individual *Stb* genes are not currently effective against *Z*. *tritici* populations in Europe (see Section 5.1; also Arraiano et al., 2009) and have not been durable (Section 5.2), although some are associated with minor quantitative resistance (Section 4.1). The majority of variation in field resistance to STB, therefore, must be controlled by quantitative resistance, as defined in Section 1, and the progress in breeding for STB-resistance over the last 30 years presumably happened by the gradual accumulation of minor genes (Torriani et al., 2015). This type of resistance appears to be more durable than qualitative resistance. Its effectiveness may be gradually eroded (Mundt et al., 2002; Krenz et al., 2008) but this happens much more slowly and to a lesser extent than the rapid evolution of virulence in a gene-for-gene interaction (Poland et al., 2008; Brown, 2015).

A significant problem in the genetics of STB is that, when a gene has a large effect on resistance to the current pathogen population and therefore seems desirable as a source of resistance in breeding, there is currently no way of determining from its phenotype or underlying mechanism whether or not it might be durable. Qualitative genes which control detection of a specific pathogen genotype in a gene-for-gene relationship are much less likely to be durable than those that enhance downstream defences. The latter class of gene includes *Lr34* in wheat against biotrophic pathogens (Krattinger et al., 2009), *STV11* in rice against *Rice stripe virus* (Wang et al., 2014) and several others. In rusts and powdery mildews, gene-for-gene interactions generate a hypersensitive response and reduce the infection type (IT) of pustules or colonies, whereas quantitative resistance tends to reduce the extent of symptoms rather than the IT (Boyd et al., 1995; Jagger et al., 2011). No such distinction can yet be made between the phenotypes of genotype-specific qualitative resistance and other, potentially more durable forms of resistance to STB. As *Z*. *tritici* is initially endophytic, becoming necrotrophic in its pathogenic phase (Orton et al., 2011; Sánchez-Vallet et al., 2015), the only distinction between compatible and incompatible interactions known at present is the amount of disease visible on the leaf (Chartrain et al., 2004b).

If a plant resistance gene introduced from a wild population or a genetically diverse landrace population is effective against the current pathogen population, it will not necessarily be durable. If it follows the gene-for-gene relationship, virulence may be rare because the resistance gene has a fitness cost or the disease is not severe at the place of origin of the resistance gene (Brown and Tellier, 2011). *Stb16q* is intriguing and potentially useful because it has a strong effect against all the large number of *Z*. *tritici* isolates with which it has been tested (Tabib Ghaffary et al., 2012). Until it is isolated, however, or unless an isolate virulent to *Stb16q* is discovered, it will not be possible to tell whether it controls a gene-for-gene resistance which is effective against the current pathogen population, or is part of the plant’s downstream defences and therefore may be durable.

Greater knowledge about mechanisms of STB-resistance would support wheat breeding, particularly by characterising the difference between gene-for-gene resistance and other kinds of resistance which may be more durable. This would help breeders to make informed decisions about the likely durability of resistance without the lengthy process of isolating the gene. A method of selecting both effective qualitative resistance, which may not be durable, and a good level of quantitative, possibly durable resistance in the same cultivar would be especially useful (Risser et al., 2011).

In addition to disease resistance, STB levels can also be reduced by traits that contribute to disease escape, which limits the spread of fungal inoculum within crops (van Beuningen and Kohli, 1990; Simon et al., 2004b; Arraiano et al., 2009). This typically happens in cultivars which are taller and later-heading, as both traits reduce the spread of spores to the upper leaves. Escape traits can be undesirable, however, because they can be maladaptive in terms of agronomic properties and yield.

A general approach to increasing quantitative resistance (to any disease) stems from viewing plant breeding as a greatly accelerated form of natural selection, in which variation in traits is selected by breeders and inherited by the next generation of cultivars. The three essential requirements for breeding for effective, durable STB-resistance are diverse germplasm, efficient breeding processes which generate new combinations of genes, and field trial sites with high levels of STB at which resistant cultivars with good agronomic properties can be selected reliably and consistently. Once these fundamentals are in place, targetted selection of cost-effective genes or combinations of genes (Grimmer et al., 2015; Torriani et al., 2015) can contribute to raising the level of STB-resistance in new wheat cultivars.

## Figures and Tables

**Table 1:** Major genes for resistance of bread wheat (*Triticum aestivum*) to *Septoria tritici* blotch, with their chromosomal locations, nearest markers, *Z*. *tritici* isolates with which they were identified, growth stage at which plants were inoculated (S: seedling, A: adult) and resistant source line.

Gene	Chromo-some	Associated markers (distance to gene)	Avirulent inoculum	Stage	Resistance source	References
Stb1	5BL	Xbarc74 (2.8cM), Xgwm335 (7.4cM)	IN95-Lafayette-1196-WW 1-4 & Purdue local (USA)	S, A	Bulgaria 88	Adhikari et al. (2004a)
Stb2	1BS	Xwmc406 (6cM), Xwmc230 (5cM)	Paskeville local (Australia) (and IPO92034)	A	Veranopolis	Liu et al. (2013)
Stb3	7AS	Xwmc83	Paskeville local isolate (Australia)	A	Israel 493	Goodwin and Thompson (2011)
Stb4	7DS	Xgwm111 (0.7cM)	IN95-Lafayette-1196-WW-1-4, I-89, IPBr1	S, A	Tadinia	Adhikari et al. (2004c)
Stb5	7DS	Xgwm44 (7.2cM)	IPO94269	S, A	Synthetic 6x	Arraiano et al. (2001b)
Stb6	3AS	Xgwm369 (2cM)	IPO323	S, A	Flame, Hereward	Brading et al. (2002)
Stb7	4AL	Xwmc313 (0.3 to 0.5cM), Xwmc219 (1cM)	MG2 (Canada) (and IPO87019)	S	ST6	McCartney et al. (2003)
Stb8	7BL	Xgwm146 (3.5cM), Xgwm577 (5.3cM)	IN95-Lafayette-1196-WW 1-4	A	Synthetic W7984	Adhikari et al. (2003)
Stb9	2BL	Xfbb226 (3.6cM), Xwmc317, Xbarc0129	IPO89011	S	Courtot, Tonic	Chartrain et al. (2009)
Stb10	1Dc	Xgwm848	IPO94269 and ISR8036	S	Kavkaz-K4500	Chartrain et al. (2005c)
Stb11	1BS	Xbarc008 (1cM)	IPO90012	S	TE9111	Chartrain et al. (2005a)
Stb12	4AL	Xwmc219	ISR398 and ISR8036	S	Kavkaz-K4500	Chartrain et al. (2005c)
Stb13	7BL	Xwmc396 (7-9cM)	MG96-36, MG2 (Canada)	S	Salamouni	Cowling (2006)
Stb14	3BS	Xwmc500 (2cM), wmc632 (5cM)	MG2 (Canada)	S	Salamouni	Cowling (2006)
Stb15	6AS	Xpsr904 (14cM)	IPO88004	S	Arina, Riband	Arraiano et al. (2007b)
StbSm3	3AS	barc321 (1.9cM)	MG96-36, MG2 (Canada)	S	Salamouni	Cuthbert (2011)
Stb16q	3DL	Xgwm494 (4.3cM), Xbarc128 (9.9cM)	IPO88018 and IPO94218	S, A	SH M3	Tabib Ghaffary et al. (2012)
Stb17	5AL	Xhbg247 (3.1cM), Xgwm617 (38.3cM)	IPO88018	A	SH M3	Tabib Ghaffary et al. (2012)
Stb18	6DS	Xgpw5176, Xgpw3087	IPO323, IPO98022, IPO89011, IPO98046	S, A	Balance	Tabib Ghaffary et al. (2011)
StbWW	1BS	Xbarc119b (0.9–4.1cM)	79, 2, 1A	S	WW1842, WW2449, WW2451	Raman et al. (2009)
TmStb1	7A^m^S	Xbarc174 (23.5cM)	IPO323	S	MDR043 (T. monococcum)	Jing et al. (2008)
